# The health-economic impact of urine albumin-to-creatinine ratio testing for chronic kidney disease in Japanese non-diabetic patients

**DOI:** 10.1007/s10157-024-02600-9

**Published:** 2024-12-16

**Authors:** Tsuneo Konta, Koichi Asahi, Kouichi Tamura, Fumitaka Tanaka, Akira Fukui, Yusuke Nakamura, Junichi Hirose, Kenichi Ohara, Yoko Shijoh, Matthew Carter, Kimberley Meredith, James Harris, Örjan Åkerborg, Naoki Kashihara, Takashi Yokoo

**Affiliations:** 1 Department of Public Health and Hygiene, Yamagata University Graduate School of Medicine, Yamagata, Japan; 2 Division of Nephrology and Hypertension, Department of Internal Medicine, Iwate Medical University School of Medicine, Yahaba, Japan; 3 Department of Medical Science and Cardiorenal Medicine, Yokohama City University Graduate School of Medicine, Yokohama, Japan; 4 Division of Nephrology and Hypertension, Department of Internal Medicine, The Jikei University School of Medicine, Minato City, Japan; 5 Bayer Yakuhin Ltd, Osaka, Japan; 6 Wickenstones Ltd, Carlow, Ireland; 7 Kawasaki Geriatric Medical Center, Okayama, Japan

**Keywords:** Albuminuria, Cost-effectiveness analysis, Japan, Renal insufficiency, Chronic

## Abstract

**Background:**

The objective of this analysis was to estimate the clinical and economic impact of undertaking urine albumin-to-creatinine ratio (UACR) testing alongside regular estimated glomerular filtration rate testing for chronic kidney disease in non-diabetic Japanese patients versus no testing and versus urine protein-creatinine ratio (UPCR) testing.

**Methods:**

An economic model, taking a Japanese healthcare perspective, estimated the health-economic impact of UACR testing over a lifetime time horizon. Outcomes reported were additional costs, clinical benefits measured, such as prevented dialyses and cardiovascular events, quality-adjusted life years gained, and incremental cost-effectiveness ratios. Health states were derived from risk levels reported in the Kidney Disease: Improving Global Outcomes heatmap. Results were derived assuming that after testing, treatment was available in the form of current standard-of-care or emerging chronic kidney disease therapies.

**Results:**

Repeated UACR testing was found to be cost-effective compared to both no urine testing and UPCR testing, with incremental cost-effectiveness ratios of ¥1,953,958 and ¥1,966,433, respectively.

**Conclusion:**

Overall, this model demonstrates the health-economic value of undertaking UACR testing within the non-diabetic Japanese population.

**Supplementary Information:**

The online version contains supplementary material available at 10.1007/s10157-024-02600-9.

## Introduction

Chronic kidney disease (CKD) is a progressive condition and is among the leading causes of mortality worldwide [1]. In Japan, the estimated prevalence of CKD in the general population is between 10 and 13% [2, 3]. However, due to the early stages of CKD being asymptomatic, nearly half go undiagnosed [4]. Until recently, patients lacked efficacious CKD therapies and were faced with irreversible and inevitable kidney damage, leading to end-stage kidney disease (ESKD) and a requirement for kidney replacement therapy [5].

Globally, CKD management is a strong contributing factor to rising healthcare expenditures, with CKD contributing more than other high-burden diseases like type 2 diabetes and chronic obstructive pulmonary disease [6, 7]. These costs are further exacerbated by aging populations and more patients progressing to ESKD. In addition, the proportion of people with CKD not explained by diabetes or hypertension is continuing to grow in developing countries, particularly in younger patients [8].

The rising prevalence and burden of CKD highlight the urgent need for more proactive measures for early detection and treatment to slow CKD progression. Early diagnosis allows patients to adjust their lifestyle with regular exercise, smoking cessation, and a low-protein diet while addressing underlying health risks like hypertension [9]. Furthermore, patients can receive therapies that can directly slow and even inhibit the progression of CKD [10], reducing the likelihood of reaching ESKD [11–13].

For the identification of CKD, the amount of kidney damage and the level of kidney function decline needs to be assessed. Patients with CKD may have significant kidney damage but still have normal kidney function, requiring consideration of both factors for an accurate diagnosis [14–18].

Global guidelines recommend that kidney function should be evaluated using an estimated glomerular filtration rate (eGFR) test and kidney damage using the following tests, in descending preference: urine albumin-to-creatinine (UACR) tests, urine protein-to-creatinine ratio (UPCR) tests, and dipstick tests [19, 20]. Furthermore, UACR testing is reimbursed globally for non-diabetic CKD treatment in populations with risk factors, such as hypertension, prior acute kidney injuries, and cardiovascular disease [21].

Japanese guidelines [22] differ in their discussions of UACR, UPCR, and dipstick testing in relation to the Japanese reimbursement situation. In consideration of test characteristics and costs, the Ministry of Health, Labour and Welfare (MHLW) has established the following criteria for reimbursement: dipstick testing is used for health check-ups and CKD screening, UPCR for advanced diabetic kidney disease and non-diabetic CKD treatment, and UACR for early diagnosis and prediction of diabetic kidney disease [23]. Thus, UPCR testing is utilized more [24] than UACR testing in the non-diabetic Japanese patient population due to being reimbursed, despite having lower accuracy for determining kidney damage.

The hypothesis for this study is that UACR testing is cost-effective in the non-diabetic population in Japan, which has been demonstrated in other high-risk populations within existing literature [25]. To date, there have been no cost-effectiveness analyses, from the authors’ knowledge, to show the value of UACR testing compared to UPCR testing in a non-diabetic population globally. Therefore, an economic model was created to demonstrate the value of regularly measuring kidney damage with a UACR test versus no testing, and versus UPCR testing, in the non-diabetic Japanese population.

## Materials and methods

### Overview

A cost-effectiveness analysis was undertaken in Microsoft Excel to evaluate the health-economic impact of regularly measuring kidney damage with a UACR test alongside regular eGFR testing in the non-diabetic Japanese population, by answering the following research questions:

1. What is the value of regularly testing for kidney damage with a UACR test versus not testing for kidney damage?
2. What is the value of regularly testing for kidney damage with a UACR test versus testing with a UPCR test?

The analysis took the perspective of the Japanese healthcare system. Model parameters were captured from an English-language and Japanese-language systematic review (SR) of the Japanese non-diabetic population (Online Resource 1), aggregate data from health check-up participants in Yamagata and Iwate prefectures [26, 27], targeted searching, and Japanese medical fee schedule tables [28, 29]. Benefits were reported as quality-adjusted life years (QALYs) gained and dialyses prevented, and the primary outcomes were reported as ICERs. Costs and benefits were discounted by 2% per annum, as per Japanese guideline recommendations [30]. Costs were captured and reported in 2023 Japanese Yen.

### Decision tree

**Fig. 1:**
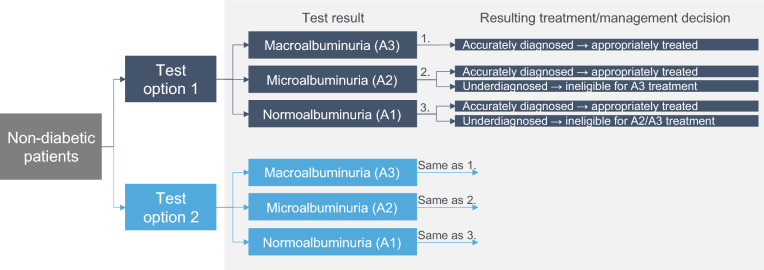
Decision tree exploring the diagnosis and subsequent treatment/management resulting from different tests for the identification of kidney damage. Test options 1 and 2 can be either UACR, UPCR, or no-testing, depending on the comparison of interest. Where UPCR testing is selected, the test results instead refer to the corresponding proteinuria categories: macroalbuminuria corresponds to severely increased proteinuria, microalbuminuria corresponds to mildly/moderately increased proteinuria, and normoalbuminuria corresponds to normal to mildly increased proteinuria. *Abbreviations:* CKD, chronic kidney disease; UACR, urine albumin-to-creatinine ratio; UPCR, urine protein-creatinine ratio

A decision tree (Fig. 1) was constructed to explore the comparative impact of UACR testing versus no testing and UACR testing versus UPCR testing on diagnosis and subsequent treatment/management strategies. It was assumed that patients in both arms received regular eGFR testing. For the no urine testing cohort, albuminuria would not be identified, and therefore any treatment or management decisions would be made under the assumption of normal albuminuria levels (A1). To directly compare results between UACR and UPCR tests, it was assumed that equivalent categorizations would have the same influence on treatment and management decisions. Categories considered equivalent were, for UACR and UPCR, respectively: macroalbuminuria or severely increased proteinuria, microalbuminuria or mildly/moderately increased proteinuria, and normoalbuminuria or normal to mildly increased proteinuria. To make a comparison between diagnosis with alternate tests, it was assumed that UACR results were gold standard for diagnosis [31].

Test performance across UPCR and UACR tests was contrasted within the decision tree using aggregate re-test data derived from health check-up participants in the Yamagata prefecture in Japan [26]. For each test cohort, the proportion of individuals diagnosed at each level of albuminuria was input (Table 1). Cost-effectiveness was also explored in sub-groups with prior dipstick test results (Online Resource 2).

**Table 1:** Re-test data

	Initial test			Subsequent test for reclassification		
	UPCR results			UACR results		
	~A1	~A2	~A3	A1	A2	A3
Proportion of cohort	0.929 (*n* = 1150)	0.061^a^ (*n* = 75)	0.011^a^ (*n* = 13)	0.830 (*n* = 1027)	0.160 (*n* = 198)	0.011 (*n* = 13)

	Dipstick result			UACR results		
	Negative			A1	A2	A3
Proportion of cohort	1.0 (*n* = 1109)			0.892 (*n* = 989)	0.108 (*n* = 120)	0.000 (*n* = 0)

	Dipstick result			UACR results		
	Trace			A1	A2	A3
Proportion of cohort	1.0 (*n* = 69)			0.406 (*n* = 28)	0.594 (*n* = 41)	0.000 (*n* = 0)

	Dipstick result			UACR results		
	Positive			A1	A2	A3
Proportion of cohort	1.0 (*n* = 60)			0.167 (*n* = 10)	0.617 (*n* = 37)	0.217 (*n* = 13)

	Dipstick result			UPCR results		
	Negative			~A1	~A2	~A3
Proportion of cohort	1.0 (*n* = 1109)			0.956 (*n* = 1060)	0.044^b^ (*n* = 49)	0.000^b^ (*n* = 0)

	Dipstick result			UPCR results		
	Trace			~A1	~A2	~A3
Proportion of cohort	1.0 (*n* = 69)			0.812 (*n* = 56)	0.188 (*n* = 13)	0.000 (*n* = 0)

	Dipstick result			UPCR results		
	Trace			~A1	~A2	~A3
Proportion of cohort	1.0 (*n* = 60)			0.567 (*n* = 34)	0.267 (*n* = 16)	0.167 (*n* = 10)

Source: Aggregate data from health check-up participants in the Yamagata prefecture in Japan

^a^ Original data included 1 patient classified as severely increased proteinuria that would have been classified as A2 with a UACR test, so for this analysis they were moved to ~ A2, as it was assumed that patients could not be diagnosed more severely with a UPCR test than was representative of their true underlying CKD risks

^b^ Original data included 4 patients classified as severely increased proteinuria after a negative dipstick test result; however, they were moved to ~ A2 for the same reason as above

Abbreviations: UACR, urine albumin-to-creatinine ratio; UPCR, urine protein-creatinine ratio

### Markov model

A decision analytic Markov cohort model was adapted from a previously conducted analysis taking a UK healthcare perspective [32]. This allowed for a health-economic analysis of the alternate testing strategies and subsequent treatment/management decisions. Sensitivity analysis was performed to ascertain drivers and evaluate stability of the results (Online Resource 3).

**Fig. 2:**
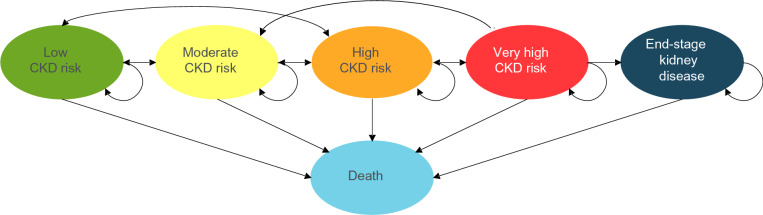
Health state diagram for patient flow within Markov model. *Abbreviations:* CKD, chronic kidney disease

The model structure comprised six mutually exclusive health states, informed by the stages of CKD risk outlined in the KDIGO guidelines [31]. These health states (Fig. 2) were low risk of CKD, moderate risk of CKD, high risk of CKD, very high risk of CKD, ESKD, and death. The starting distribution of patients across these health states was informed by aggregate data from health check-up participants in the Yamagata prefecture in Japan [26] (Table 2). A cycle length of one year was employed, with a starting age of 60 across the cohort and a lifetime horizon (maximum patient age of 100).

**Table 2:** Yamagata aggregate data population characteristics. *Source:* Aggregate data from health check-up participants in the Yamagata prefecture in Japan

Parameter	Base-case input value
Patient numbers, *n*	1238
Age, years ± s.d	63.4 ± 10.0
Female, *n* (%)	578 (46.7)
Hypertension, *n* (%)	581 (47.0)
*eGFR*	
G1, %	28.5
G2, %	64.2
G3a, %	6.6
G3b, %	0.4
G4, %	0.2
G5, %	0.1
*UACR*	
Normoalbuminuria, %	83
Microalbuminuria, %	16
Macroalbuminuria, %	1.1

eGFR, estimated glomerular filtration rate; s.d., standard deviation; UACR, urine albumin-to-creatinine ratio

Each cohort of patients progressed through the model at different rates, resulting in the UACR tested cohort having slower progression throughout the model. This was driven by the different numbers of patients eligible for treatment due to a test result indicating kidney damage. The base case analysis assumes patients diagnosed in KDIGO categories G1:G4, A2:A3 were eligible for treatment with angiotensin-converting enzyme inhibitors/angiotensin II receptor blockers (ACEi/ARBs). Whilst not explicitly included in the Japanese guidelines for CKD, expert consultations with research members from the Japan Kidney Association confirmed that ACEi/ARBs are actively used for CKD patients and are considered off-label standard of care in Japan. Therefore, ACEi/ARBs were included within the model to best represent the current clinical practice. It was assumed that patients with comparable diagnoses would receive the same treatment/management regardless of testing strategy.

### Transition probabilities

Two different approaches were applied to estimate the transition probabilities between health states. For the starting health state, parameters used were based on categories in the KDIGO heatmap [31]. For subsequent transitions, parameters were used based on the average risk for the respective health state.

Transition probabilities for the second approach were generated by a subset of data from the Yamagata aggregate data (Table 2). Where data gaps were present, expert assumptions were used. Transition probabilities, prior to adjustment for treatment, can be found in Table 3. Transition probabilities for the first approach were generated by multiplying the health state transition probabilities from the second approach by adjustment factors (Table 4) derived from the relationship between KDIGO categories and risk of progressive CKD demonstrated by global meta-analyses [33].

**Table 3:** Transition probabilities

Parameter	Base-case input value	Source
Low CKD risk to moderate CKD risk	0.085	Yamagata aggregate data
Low CKD risk to high CKD risk	0.002	
Low CKD risk to very high CKD risk	0.000	
Low CKD risk to ESKD	0.000	
Moderate CKD risk to low CKD risk	0.337	
Moderate CKD risk to high CKD risk	0.067	
Moderate CKD risk to very high CKD risk	0.000	
Moderate CKD risk to ESKD	0.000	
High CKD risk to low CKD risk	0.067	
High CKD risk to moderate CKD risk	0.267	
High CKD risk to very high CKD risk	0.067	
High CKD risk to ESKD	0.000	
Very high CKD risk to low CKD risk	0.000	Assumption
Very high CKD risk to moderate CKD risk	0.010	
Very high CKD risk to high CKD risk	0.040	
Very high CKD risk to ESKD	0.100	
ESKD to low CKD risk	0.000	
ESKD to moderate CKD risk	0.000	
ESKD to high CKD risk	0.000	
ESKD to very high CKD risk	0.000	

CKD, chronic kidney disease; ESKD, end-stage kidney disease

**Table 4:** Transition probability adjustment factors

KDIGO category	Transition probability adjustment factor	Source
G1A1	1	Assumption based on [33]
G1A2	0.45	
G1A3	0.42	
G2A1	1	
G2A2	1.14	
G2A3	0.90	
G3aA1	1.21	
G3aA2	1.26	
G3aA3	0.9	
G3bA1	0.99	
G3bA2	0.63	
G3bA3	1.2	
G4A1	0.40	
G4A2	1	
G4A3	1.5	

KDIGO, Kidney Disease: Improving Global Outcomes

Background mortality rates from Japanese life expectancy tables [34] were used in conjunction with hazard ratios for mortality by CKD stage [35] identified within the SR to estimate mortality risks (Table 5). The model also captured the incidence of cardiovascular events based on incidence rates for reference group patients [20, 36] and hazard ratios for each stage of CKD (Table 6).

**Table 5:** Hazard ratio for mortality

Parameter	Base-case input value	Source
G1A1	1.140	[35]
G1A2	1.880	
G1A3	4.010	
G2A1	1.140	
G2A2	1.790	
G2A3	2.130	
G3aA1	1.710	
G3aA2	2.390	
G3aA3	2.570	
G3bA1	2.590	
G3bA2	3.820	
G3bA3	4.030	
G4A1	4.710	
G4A2	4.410	
G4A3	5.860	
G5A1	4.850	
G5A2	12.000	
G5A3	8.690

**Table 6:** Incidence rate and hazard ratios for cardiovascular events, as well as the proportion of events by cause

CKD risk (outcome)	Risk of cardiovascular event	Source
Low risk CKD (incidence rate)	0.109	[20]
Low risk CKD (hazard ratio)	1.00	
Moderate risk CKD (hazard ratio)	1.52	
High risk CKD (hazard ratio)	2.04	
Very high-risk CKD (hazard ratio)	3.69	
ESKD risk CKD (hazard ratio)	12.00	Expert input

Cardiovascular events	Proportion of cardiovascular events	Source
Myocardial infarction	0.09	Iwate aggregate data ^a^
Stroke (ischemic)	0.63	Iwate aggregate data ^a^, with rate of stroke type informed by [36]
Stroke (intracerebral hemorrhage)	0.15	Iwate aggregate data ^a^, with rate of stroke type informed by [36]
Stroke (subarachnoid hemorrhage)	0.06	Iwate aggregate data ^a^, with rate of stroke type informed by [36]
Other CV event	0.07	Iwate aggregate data ^a^

CKD, chronic kidney disease; CV, cardiovascular

^a^ Aggregate data from health check-up participants in the Iwate prefecture in Japan

The base case analyzed relative risk reduction for ACEi/ARBs (for patients in categories G1-4, A2-3); however, as the benefits of early diagnosis depend on the treatment available following diagnosis, an additional scenario was analyzed. This additional scenario analyzed a combination therapy using ACEi/ARBs (G1-4, A2-3) and sodium–glucose cotransporter 2 inhibitors (SGLT2is; G3a-4, A2 or G1-4, A3). Relative risk reductions for ACEi/ARBs and ACEi/ARBs + SGLT2is (Table 7) [37, 38] were identified through targeted searching and applied to the transition probabilities of patients eligible for treatment.

**Table 7:** Treatment effects

Treatment	Relative risk reductions			
	Progression	Source	Mortality	Source
ACEi/ARBs	0.21	[37]	0.1	[37]
ACEi/ARBs and SGLT2is	0.35	[38]	0.1	Assumption

ACEi/ARBs, angiotensin-converting-enzyme inhibitors/angiotensin receptor blockers; SGLT2is, sodium–glucose cotransporter 2 inhibitors

### Other parameters

Costs used within the model were taken from Japanese medical fee schedule tables and literature sources [28, 29, 39, 40]. Costs from both sources were then validated by Japanese clinicians (Table 8). Annual resource use rates per health state were suggested by Japanese clinicians (Table 9). Published costs from previous years were inflated based on annual consumer price indexes for Japan [41], up to 2022. For costs associated with cardiovascular events, only costs of care for acute events were considered.

**Table 8:** Costs (by health state)

Cost category	Cost (¥)	Source
*Annual treatment costs*		
ACEi/ARBs	4015.00	Medical Data Vision database [39] and expert validation
SGLT2is	96,725.00	Japanese medical fee schedule table [29] and expert validation
Dialysis	5,456,714.14	[40] (adjusted for inflation) & expert validation
*Unit costs*		
GP consultation	730.00	Japanese medical fee schedule table [28] and expert validation
GP tests (Low risk)	2870.00	
GP tests (Moderate risk)	480.00	
GP tests (High risk)	960.00	
GP tests (Very high risk)	0.00	
GP prescription (Low risk)	0.00	
GP prescription (Moderate risk)	3180.00	
GP prescription (High risk)	0.00	
GP prescription (Very high risk)	0.00	
Nephrologist consultation	740.00	
Nephrologist tests (Low risk)	0.00	
Nephrologist tests (Moderate risk)	5370.00	
Nephrologist tests (High risk)	11,460.00	
Nephrologist tests (Very high risk)	12,670.00	
Nephrologist prescription (Low risk)	0.00	
Nephrologist prescription (Moderate risk)	0.00	
Nephrologist prescription (High risk)	30,680.00	
Nephrologist prescription (Very high risk)	24,740.00	
Other nephrologist costs (Low risk)	0.00	
Other nephrologist costs (Moderate risk)	2000.00	
Other nephrologist costs (High risk)	2000.00	
Other nephrologist costs (Very high risk)	10,000.00	
eGFR test	110.00	
UACR test	1730.00	
UPCR test	810.00	
*One-time event/surgery costs*		
Surgery for vascular access	1,579,488.00	Japanese medical fee schedule table [28] and expert validation
Myocardial Infarction	3,090,988.00	
Stroke (Ischemic)	2,491,671.00	
Stroke (Intracerebral hemorrhage)	5,156,209.00	
Other CV event	2,214,259.00	

ACEi/ARBs, angiotensin-converting enzyme inhibitors/angiotensin II receptor blockers; eGFR, estimated glomerular filtration rate; GP, general physician; SGLT2is, sodium/glucose cotransporter 2; UACR, urine albumin-to-creatinine ratio; UPCR, urine protein-creatinine ratio

**Table 9:** Annual healthcare resource utilization by health state. Source: All resource use values were based on Japanese expert input

Resource use category	Annual resource use per patient
Low risk CKD GP appointments	1
Moderate risk CKD GP appointments	12
High risk CKD GP appointments	12
Very high-risk CKD GP appointments	0
Low risk CKD nephrologist appointments	0
Moderate risk CKD nephrologist appointments	2
High risk CKD nephrologist appointments	4
Very high-risk CKD nephrologist appointments	12
Low risk CKD eGFR tests	1
Moderate risk CKD eGFR tests	4
High risk CKD eGFR tests	8
Very high-risk CKD eGFR tests	4
ESKD eGFR tests	4
Low risk CKD UACR tests	1
Moderate risk CKD UACR tests	2
High risk CKD UACR tests	4
Very high-risk CKD UACR tests	4
ESKD UACR tests	4
Low risk CKD UPCR tests	1
Moderate risk CKD UPCR tests	2
High risk CKD UPCR tests	4
Very high-risk CKD UPCR tests	4
ESKD UPCR tests	4

CKD, chronic kidney disease; eGFR, estimated glomerular filtration rate; GP, general physician; UACR, urine albumin-to-creatinine ratio; UPCR, urine protein-creatinine ratio

Utility values per KDIGO category were not identified by the SR, hence values by eGFR category were identified through additional targeted searching [42], and utility values for the model health states (Table 10) were calculated via weighted averages. For each health state, this was calculated as the utility value for each eGFR category weighted by the proportion of patients in each eGFR category.

**Table 10:** Utility values for health states

Parameter	Base-case input value	Source
Low CKD risk	0.940	[42] weighted by Yamagata aggregate data ^a^
Moderate CKD risk	0.913	
High CKD risk	0.900	
Very high CKD risk	0.859	
ESKD	0.789	

CKD, chronic kidney disease; ESKD, end-stage kidney disease

^a^ Aggregate data from health check-up participants in the Yamagata prefecture in Japan

## Results

### UACR testing versus no urine testing

Testing for kidney damage with regular UACR testing yielded an overall QALY gain in the base case of 32.90, producing an ICER of ¥1,953,958. This value should be contrasted to the commonly cited willingness-to-pay cut-offs of ¥5,000,000 for the health technology assessment (HTA) process in Japan, a threshold that achieved consensus by the Central Social Insurance Medical Council (Chuikyo) [43]. Per 1000-patient cohort, treating with ACEi/ARBs led to an additional cost of ¥64,288,914 (Table 11).

**Table 11:** UACR versus no testing clinical, cost, and cost-effectiveness results

Test comparator	UACR	No testing	Difference
*No preceding dipstick test*			
Cost (¥)	1,750,743,334	1,686,454,420	64,288,914
CV events	335.68	336.29	− 0.61
Dialysis	18.06	19.13	− 1.08
LYs	19,274.39	19,241.21	33.18
QALYs	17,977.67	17,944.77	32.90
ICER (¥ / QALYs gained)			1,953,958

Results presented for a cohort of 1000 individuals

CV, cardiovascular; ICER, incremental cost-effectiveness ratio; LYs, life years; QALYs, quality-adjusted life years; UACR, urine albumin-to-creatinine ratio

### UPCR testing versus no urine testing

Results for regular UPCR versus no testing followed the same trend (Table 12). The overall QALY gain over no testing amounted to 14.13, less than half that compared to UACR testing versus no testing. Additional costs totaled to ¥27,370,981 per 1000 patients. The combination of fewer QALYs gained and fewer additional costs resulted in very comparable cost-effectiveness relative to UACR testing versus no urine testing.

**Table 12:** UPCR versus no testing clinical, cost, and cost-effectiveness results

Test comparator	UPCR	No testing	Difference
*No preceding dipstick test*			
Cost (¥)	1,713,825,401	1,686,454,420	27,370,981
CV events	335.96	336.29	− 0.33
Dialysis	18.61	19.13	− 0.53
LYs	19,255.41	19,241.21	14.20
QALYs	17,958.89	17,944.77	14.13
ICER (¥ / QALYs gained)			1,937,381

Results presented for a cohort of 1000 individuals; treatment effects based on ACEi/ARBs

CV, cardiovascular; ICER, incremental cost-effectiveness ratio; LYs, life years; QALYs, quality-adjusted life years; UPCR, urine protein-creatinine ratio

### UACR testing versus UPCR testing

When testing for kidney damage, regular UACR testing is preferable compared to UPCR testing and was shown to be cost-effective (Table 13). A total of 18.77 QALYs were gained in the UACR cohort relative to the UPCR cohort, for an additional cost of ¥36,917,933 per 1000 patients.

**Table 13:** UACR versus UPCR clinical, cost, and cost-effectiveness results

Test comparator	UACR test	UPCR test	Difference
*No preceding dipstick test*			
Cost (¥)	1,750,743,334	1,713,825,401	36,917,933
CV events	335.68	335.96	− 0.29
Dialysis	18.06	18.61	− 0.55
LYs	19,274.39	19,255.41	18.98
QALYs	17,977.67	17,958.89	18.77
ICER (¥ / QALYs gained)			1,966,433

Results presented for a cohort of 1000 individuals; treatment effects based on ACEi/ARBs

CV, cardiovascular; ICER, incremental cost-effectiveness ratio; LYs, life years; QALYs, quality-adjusted life years; UACR, urine albumin-to-creatinine ratio; UPCR, urine protein-creatinine ratio

### Contrasting upcoming treatment paradigms

**Fig. 3:**
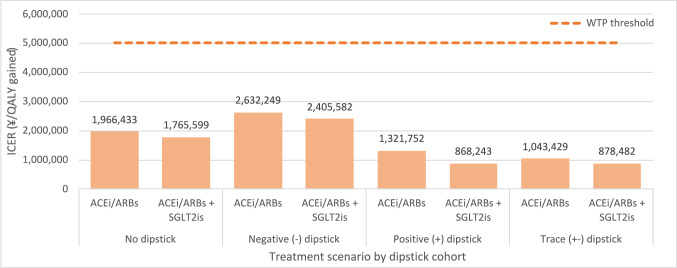
Cost-effectiveness of UACR versus UPCR by treatment paradigm. *Abbreviations*: ACEi/ARBs, angiotensin-converting enzyme inhibitors/angiotensin II receptor blockers; ICER, incremental cost-effectiveness ratio; SGLT2is, sodium–glucose cotransporter-2 inhibitors; UACR, urine albumin-to-creatinine ratio; UPCR, urine protein-creatinine ratio; WTP, willingness-to-pay

Current guidance suggests that high-risk patients should be considered for emerging, more efficacious treatment. For this reason, analysis was conducted considering treatment with a combination of ACEi/ARBs and SGLT2is. Figure 3 shows that the cost-effectiveness improves in this treatment scenario. Consequently, cost-effectiveness estimates are all improved in the emerging treatment scenario.

### Contrasting by starting risk of CKD

Cost-effectiveness of testing for kidney damage with a UACR test was found to be dominant compared to not testing for higher-risk stages of CKD. Conversely, no clinical benefit is seen in low-risk individuals where no treatment is prescribed following a diagnosis; however, it is not possible conclude individuals are low risk until a UACR test has been undertaken (Table 14).

**Table 14:** Cost and cost-effectiveness results for UACR testing versus no testing by stage of CKD

CKD stage	Outcome	UACR	No testing	Difference
Low risk	Cost (¥)	1,434,132,434	1,391,902,096	42,230,338
	QALYs	18,081.82	18,081.82	0.00
	ICER (¥ / QALYs gained)			Dominated
Moderate risk	Cost (¥)	1,961,232,030	¥1,771,557,497	189,674,533
	QALYs	17,863.52	17,732.28	131.25
	ICER (¥ / QALYs gained)			1,445,172
High risk	Cost (¥)	5,833,891,507	¥6,089,939,112	− 256,047,605
	QALYs	16,624.70	16,402.15	222.55
	ICER (¥ / QALYs gained)			Dominant
Very high risk	Cost (¥)	21,776,785,520	22,023,263,068	− 246,477,547
	QALYs	12,469.42	12,151.05	318.38
	ICER (¥ / QALYs gained)			Dominant

CKD, chronic kidney disease; ICER, incremental cost-effectiveness ratio; LYs, life years; QALYs, quality-adjusted life years; UACR, urine albumin-to-creatinine ratio

Results presented for a cohort of 1000 individuals. CKD stages are defined according to the KDIGO staging for CKD [59]

## Discussion

### Key findings

To the best of the authors’ knowledge, this is the first cost-effectiveness analysis of UACR testing compared to UPCR testing in a non-diabetic population. It demonstrates that in over-60-year-olds, measuring kidney damage with UACR testing in combination with regular eGFR testing is cost-effective compared to alternatives. A key driver for these results was the increased detection rate of microalbuminuria with a UACR test. Furthermore, a UACR test is cost-effective compared to UPCR testing. While this analysis used data for individuals older than 60 years, these findings may still have applicability to younger patients, as the effect seen is driven by the increased accuracy of UACR testing in identifying a patient’s kidney damage compared with a UPCR test.

Previous studies have found the cost-effectiveness of CKD screening approaches to vary considerably across studies [25]; however, cost-effectiveness has been particularly demonstrated in high-risk groups [44, 45]. Consistently, our results present UACR testing as a cost-effective approach.

Global guidelines widely acknowledge UACR testing in preference to UPCR testing when available, yet UPCR testing is more frequently undertaken in Japan, and UACR testing is not reimbursed in non-diabetic patients [41]. Therefore, increased accessibility to UACR testing for non-diabetic patients should become more of a priority from a public health perspective.

Benefits of UACR testing could extend towards other comorbidities that have not been considered within this analysis. High albuminuria levels are associated with a higher risk of cardiovascular disease (CVD) [46] and, therefore, early identification may present opportunities to improve patient management relative to CVD risk. Furthermore, early CKD identification through UACR and eGFR testing can reduce the chance of reaching ESKD and requiring dialysis, which may provide further benefits due to the association between dialysis and arteriosclerosis obliterans [47].

The findings for the Yamagata prefecture are believed to be generalizable to the wider population in Japan due to comparable prevalence of CKD (14%) with two other national health check-up cohorts (14.2% [48] and 16% [49]). Moreover, the Yamagata cohort baseline characteristics, including eGFR (G2), age (62.2 vs 63.6 years) and sex (39.6% vs 40.6% male), align closely with those of a nationwide screening program consisting of 332,174 individuals [48]. Whilst these findings are based upon the non-diabetic Japanese population over 60 years of age, there are many other countries with at-risk populations for which similar findings would be expected. Many countries already reimburse UACR testing for people with suspected CKD, with or without diabetes [21, 50], with countries, such as Sweden and Denmark, utilizing non-financial incentives to increase UACR testing rates [51, 52]. With increasing global rates of undiagnosed CKD, UACR testing could become a key component to overcoming this growing health concern [53].

### Limitations

Whilst efforts were taken to systematically collect robust and exhaustive data to inform the model, data gaps were identified due to a lack of literature quantifying the progression CKD across the KDIGO heatmap. Data gaps were filled using aggregate data from health check-up participants in the Yamagata and Iwate prefectures; however, this data was largely for patients in the earlier stages of CKD, meaning risk progression to very high CKD risk had to be estimated by Japanese clinicians instead. Insufficient sample sizes for risks of disease progression by KDIGO category meant that the meta-analyses forming the basis for global KDIGO guidelines were utilized [31], although this data is slightly dated (initiated in 2009).

Differences between testing modalities only impacted treatment and management up until transition from the initial model health state. Moreover, factors such as costs arising from unnecessary management of patients receiving a UPCR test have not been accounted for, hence overall, it’s possible that the cost-effectiveness of UACR may be underestimated.

Benefits of UACR testing arising outside of direct treatment effects on mortality and progression have not been incorporated in the analysis. The treatment effects used for this analysis [37, 38] were conservative relative to values used in other studies [44, 54–56]. Hence, overall, the model is likely to report a conservative estimation of the degree to which CKD can be slowed or prevented [57]. Whilst the treatment effect for combination therapy with ACEi/ARBs and SGLT2is [51] was taken from a study in diabetic patients, analyses from the EMPA-KIDNEY trial [58] have demonstrated the effects of treatment to be consistent across diabetic and non-diabetic cohorts.

### Conclusion

When considering a ≥ 60-year-old non-diabetic Japanese population, this study suggests that regular UACR testing for identification of kidney damage is cost-effective compared with no testing for kidney damage and compared with UPCR testing. Overall, this analysis demonstrates the health-economic benefits associated with UACR testing in the non-diabetic Japanese population. The findings extend their applicability on a global scale, particularly for those countries that do not prioritize UACR testing.

## Supplementary Information

Below is the link to the electronic supplementary material.

Supplementary file 1 (DOCX 90 kb)

Supplementary file 2 (DOCX 92 kb)

Supplementary file 3 (DOCX 706 kb)
