# Supplementary material for: The health-economic impact of urine albumin-to-creatinine ratio testing for chronic kidney disease in Japanese non-diabetic patients
Source: Clin Exp Nephrol. 2024 Dec 16;29(5):583–95. doi: 10.1007/s10157-024-02600-9 (PMC12049324; doi:10.1007/s10157-024-02600-9)
Supplement: Supplementary file 1 — (DOCX 90 kb) [file 10157_2024_2600_MOESM1_ESM.docx]

***Online resource 1: Systematic review***

A systematic review was conducted using two separate searches to address the following two research questions prior to conducting this analysis:

1. What is the relationship of estimated glomerular filtration rate (GFR) and albuminuria to CKD progression, mortality (including all-cause mortality and cardiovascular [CV] mortality), kidney outcomes (including end-stage kidney disease [ESKD] and CV events) and health-related quality of life (HRQoL) across the Kidney Disease Improving Global Outcomes (KDIGO) heatmap in Japanese CKD patients with and without diabetes?
   1. What is the proportion of the Japanese population in each KDIGO category?
2. What is the diagnostic accuracy of UACR, dipstick, and protein-creatinine ratio (PCR) tests in identifying CKD in Japanese patients without diabetes?

The following databases were searched as part of the systematic review:

English and Japanese language:

- Embase (Ovid interface)
- Medline (Ovid interface)
- Cochrane’s Controlled Register of Trials (CENTRAL) and the Cochrane Database of Systematic Reviews (CDSR) (Wiley interface).

Japanese language:

- ICHUSHI (Japan Medical Abstracts Society)
- CiNii articles via https://cir.nii.ac.jp/

Additionally, backward citation searching was undertaken on studies included at full-text stage to make sure all relevant studies were captured.
