# Supplementary material for: The health-economic impact of urine albumin-to-creatinine ratio testing for chronic kidney disease in Japanese non-diabetic patients
Source: Clin Exp Nephrol. 2024 Dec 16;29(5):583–95. doi: 10.1007/s10157-024-02600-9 (PMC12049324; doi:10.1007/s10157-024-02600-9)
Supplement: Supplementary file 2 — (DOCX 92 kb) [file 10157_2024_2600_MOESM2_ESM.docx]

***Online resource 2: Scenario analysis***

The impact of preceding dipstick test results on cost effectiveness of UACR testing compared to no urine testing or UPCR testing was evaluated.

Cost effectiveness of UACR testing compared to UPCR testing is most favourable in patients that had previously tested trace or positive with a dipstick test, with ICERs of ¥1,043,429 and ¥1,321,752 per QALY gained, respectively (**Table 15**). On the other hand, UACR testing remains cost effective in individuals with a negative dipstick result, with an ICER of ¥2,632,249.

**Table 15: Scenario analyses for different preceding dipstick results**

| **With preceding negative (-) dipstick** | **UACR** | **UPCR** | **Difference** |
| --- | --- | --- | --- |
| Cost (¥) | 1,668,394,813 | 1,636,550,818 | 31,843,996 |
| CV events | 333.02 | 333.20 | -0.18 |
| Dialysis | 16.56 | 16.91 | -0.35 |
| LYs | 19,295.49 | 19,283.26 | 12.23 |
| QALYs | 17,999.59 | 17,987.49 | 12.10 |
| ICER  (¥ / QALYs gained) |  |  | 2,632,249 |
| **With preceding positive (+) dipstick** | **UACR** | **UPCR** | **Difference** |
| Cost (¥) | 2,973,986,992 | 2,874,082,784 | 99,904,208 |
| CV events | 373.23 | 374.38 | -1.15 |
| Dialysis | 40.98 | 43.19 | -2.21 |
| LYs | 18,958.11 | 18,881.68 | 76.43 |
| QALYs | 17,643.99 | 17,568.40 | 75.58 |
| ICER  (¥ / QALYs gained) |  |  | 1,321,752 |
| **With preceding trace (+/-) dipstick** | **UACR** | **UPCR** | **Difference** |
| Cost (¥) | 2,010,596,814 | 1,930,586,593 | 80,010,222 |
| CV events | 345.76 | 346.93 | -1.17 |
| Dialysis | 22.18 | 24.42 | -2.24 |
| LYs | 19,210.36 | 19,132.82 | 77.54 |
| QALYs | 17,915.52 | 17,838.84 | 76.68 |
| ICER  (¥ / QALYs gained) |  |  | 1,043,429 |
| Notes: Results presented for a cohort of 1,000 individuals; treatment effects based on ACEi/ARBs. | | | |
| Abbreviations: ICER, incremental cost-effectiveness ratio; QALYs, quality-adjusted life years; UACR, urine albumin-to-creatinine ratio; UPCR, urine protein-creatinine ratio | | | |
