# Supplementary material for: The health-economic impact of urine albumin-to-creatinine ratio testing for chronic kidney disease in Japanese non-diabetic patients
Source: Clin Exp Nephrol. 2024 Dec 16;29(5):583–95. doi: 10.1007/s10157-024-02600-9 (PMC12049324; doi:10.1007/s10157-024-02600-9)
Supplement: Supplementary file 3 — (DOCX 706 kb) [file 10157_2024_2600_MOESM3_ESM.docx]

***Online resource 3: Sensitivity analysis***

Deterministic one-way sensitivity analysis (OWSA) was conducted to explore the relative importance and influence of parameters on ICER. Parameters were each varied by ± 20% compared to the base-case setting. Furthermore, to evaluate the stability and consistency of results, probabilistic sensitivity analysis (PSA) was conducted using Monte Carlo methods where selected parameters were simultaneously sampled from their respective distributions. Confidence intervals and standard deviation for inputs were largely unavailable and, therefore, confidence intervals were assumed to be ± 20% of the base-case parameter, with standard deviation estimated based on these values. Utility values were not altered during the PSA due to the differences in utility values being small between health states.

OWSA demonstrated the fluctuations in the cost effectiveness of UACR compared to UPCR assuming base-case treatment with ACEi/ARBs (**Figure 4**). The most influential drivers of cost effectiveness were the utility value of patients in G1, the cost of UACR testing, and the proportion of patients with microalbuminuria.

Probabilistic sensitivity analysis resulting from 1,000 Monte Carlo simulations is presented in **Figure 5**, whilst the cost-effectiveness acceptability curve is presented in **Figure 6**. Stability of the model is demonstrated with 96% of samples cost effective under the ¥5,000,000/QALY threshold.

**Figure 4: Tornado diagram of twelve most deviating parameters under OWSA of UACR versus UPCR**


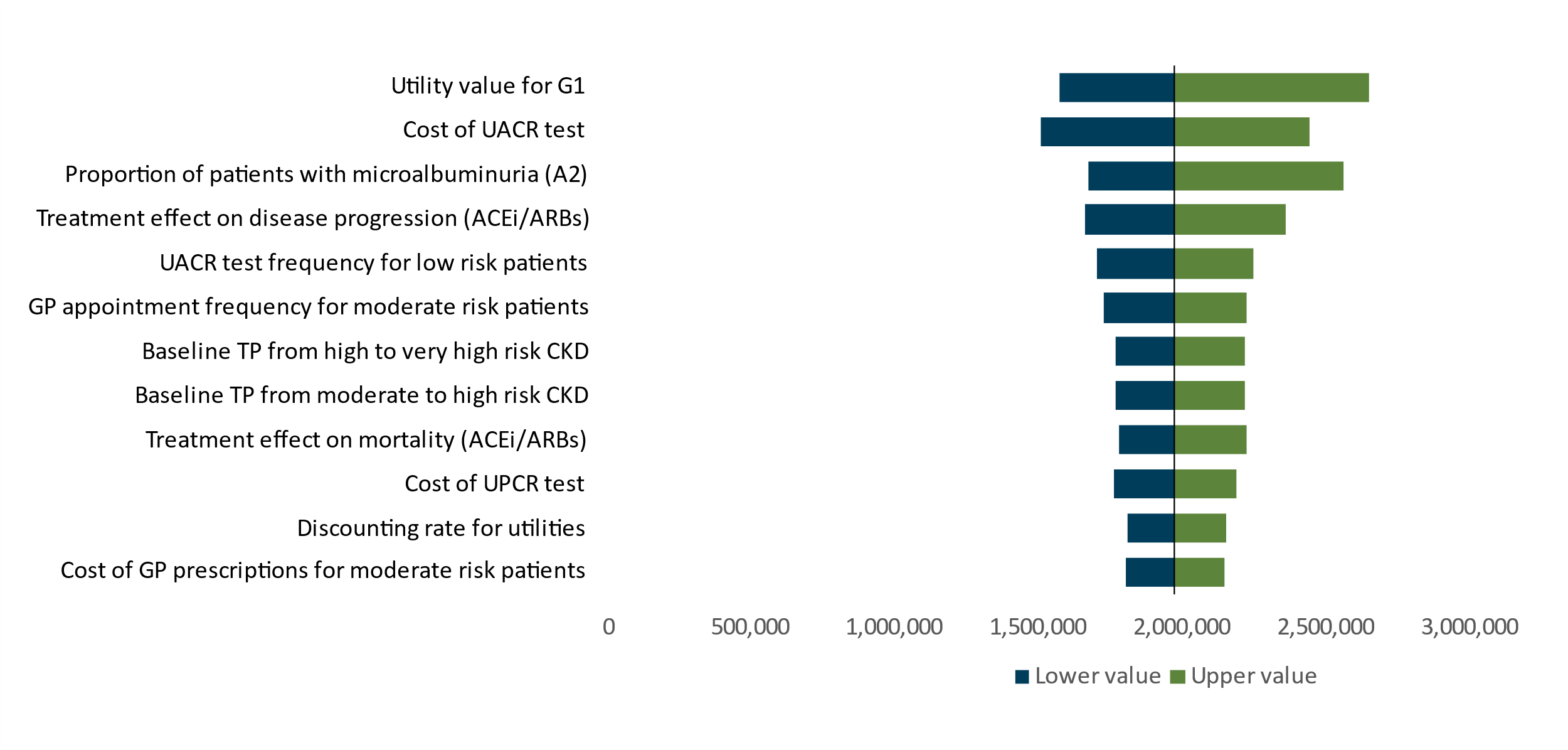


Abbreviations: ACEi/ARBs, angiotensin-converting enzyme inhibitors/angiotensin II receptor blockers; CKD, chronic kidney disease; GP, general physician; ICER, incremental cost-effectiveness ratio; OWSA, one way sensitivity analysis; TP, transition probability; UACR, urine albumin-to-creatinine ratio; UPCR, urine protein-creatinine ratio

**Figure 5: Cost-effectiveness acceptability curve of UACR versus UPCR**
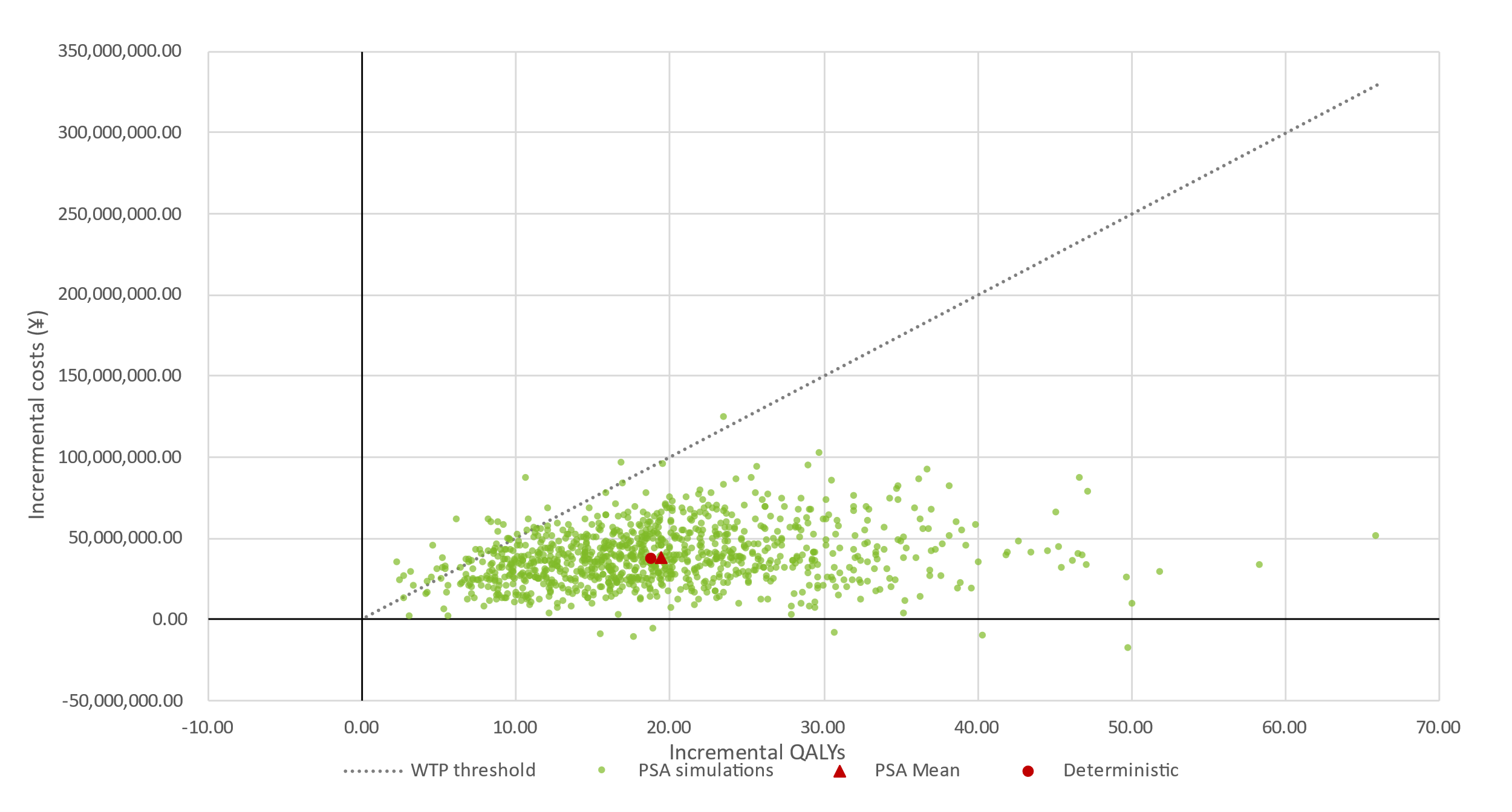


Abbreviations: PSA, probabilistic sensitivity analysis; QALYs, quality-adjusted life years; UACR, urine albumin-to-creatinine ratio; UPCR, urine protein-creatinine ratio; WTP, willingness-to-pay

**Figure 6: Cost-effectiveness plane for PSA of UACR versus UPCR**
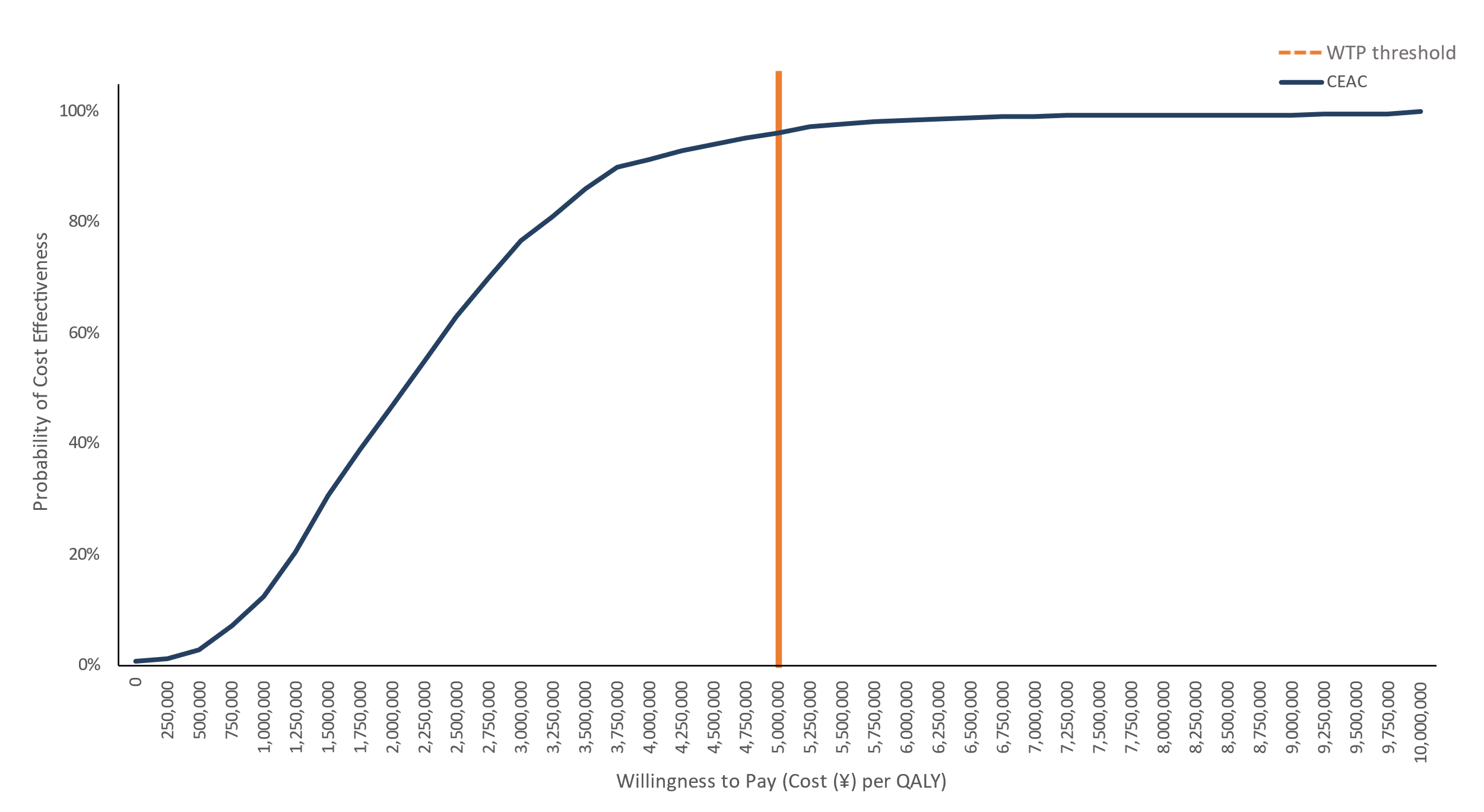
Abbreviations: CEAC, cost-effectiveness acceptability curve; PSA, probabilistic sensitivity analysis; QALY, quality-adjusted life year; UACR, urine albumin-to-creatinine ratio; UPCR, urine protein-creatinine ratio.
